# Genome Sequence of *Enterococcus faecium* Strain LCT-EF297, Which Has Specific Biochemical Features

**DOI:** 10.1128/genomeA.00102-14

**Published:** 2014-02-20

**Authors:** Li An, Yinhu Li, Zhenhong Chen, De Chang, Xuelin Zhang, Yinghua Guo, Junfeng Wang, Tianzhi Li, Wenkui Dai, Changting Liu

**Affiliations:** a Nanlou Respiratory Diseases Department, Chinese PLA General Hospital, Beijing, China; b BGI-Shenzhen, Shenzhen, China

## Abstract

An *Enterococcus faecium* strain was sent into space on the Shenzhou-VIII craft. After space flight, the strain *E. faecium* LCT-EF297 was selected based on its metabolic properties.

## GENOME ANNOUNCEMENT

An *Enterococcus faecium* strain was selected from a clinical environment. It was sent into space on the Shenzhou-VIII craft for 298 h (1). After space flight, biochemical features were determined using Biolog assays, and the strain *E. faecium* LCT-EF297 was selected for sequencing.

The genome of *E. faecium* strain LCT-EF297 was sequenced using a HiSeq 2000 (Illumina, San Diego, CA) at the Beijing Genomics Institute (BGI) (Shenzhen, China). Two randomized paired-end libraries, with average insert sizes of 500 bp and 6,000 bp, were constructed from LCT-EF297 genomic DNA. This sequencing generated 350 Mb and 200 Mb high-quality filtered data, respectively, with an average read length of 90 bp. A total of 92 contigs were obtained using SOAP*denovo* version 1.05 and were assembled on 15 scaffolds based on the paired-end relationships of reads from the large-insert library. Based on the draft genome of strain LCT-EF297, the protein-coding regions were predicted using Glimmer (version 3.02), and the gene functions were determined by BLASTp analysis with Gene Ontology (GO), KEGG, Swiss-Prot, and Clusters of Orthologous Groups (COG) databases. In addition, the sequences of rRNA, tRNA, and tandem repeats were predicted.

The draft genome of *E. faecium* strain LCT-EF297 was composed of 2,668,148 bp, with a G+C content of approximately 38%. LCT-EF297 contains 2,608 predicted genes, with a total length of 2,292,063 bp, which covers 85.90% of the genome. In total, 1,550 genes were identified by the COG database, and a subset of >200 were predicted to function in carbohydrate transport and metabolism. A total of 1,463, 1,232, and 1,619 predicted genes were identified and annotated by the KEGG, Swiss-Prot, and GO databases, respectively, and 39 tRNA genes and 4 rRNA operons were also identified. Finally, 35 microsatellites and 57 tandem repeats, with a total length of 4,216 bp, were predicted in the LCT-EF297 genome.

### Nucleotide sequence accession number.

This whole-genome shotgun project for *E. faecium* strain LCT-EF297 has been deposited at DDBJ/EMBL/GenBank under the accession no. APJQ00000000. The version described in this paper is the first version.
